# Temporal, Environmental, and Biological Drivers of the Mucosal Microbiome in a Wild Marine Fish, Scomber japonicus

**DOI:** 10.1128/mSphere.00401-20

**Published:** 2020-05-20

**Authors:** Jeremiah J. Minich, Semar Petrus, Julius D. Michael, Todd P. Michael, Rob Knight, Eric E. Allen

**Affiliations:** a Marine Biology Research Division, Scripps Institution of Oceanography, University of California San Diego, La Jolla, California, USA; b J. Craig Venter Institute, La Jolla, California, USA; c Center for Microbiome Innovation, University of California San Diego, La Jolla, California, USA; d Department of Pediatrics, University of California San Diego, La Jolla, California, USA; e Department of Computer Science and Engineering, University of California San Diego, La Jolla, California, USA; f Department of Bioengineering, University of California San Diego, La Jolla, California, USA; g Division of Biological Sciences, University of California, San Diego, La Jolla, California, USA; University of Wisconsin—Madison

**Keywords:** fish microbiome, mackerel, microbial ecology, microbial biogeography, *Scomber japonicus*, marine microbiology, veterinary microbiology

## Abstract

Changing ocean conditions driven by anthropogenic activities may have a negative impact on fisheries by increasing stress and disease. To understand how environment and host biology drives mucosal microbiomes in a marine fish, we surveyed five body sites (gill, skin, digesta, gastrointestinal tract [GI], and pyloric ceca) from 229 Pacific chub mackerel, Scomber japonicus, collected across 38 time points spanning 1 year from the Scripps Institution of Oceanography Pier (La Jolla, CA). Mucosal sites had unique microbial communities significantly different from the surrounding seawater and sediment communities with over 10 times more total diversity than seawater. The external surfaces of skin and gill were more similar to seawater, while digesta was more similar to sediment. Alpha and beta diversity of the skin and gill was explained by environmental and biological factors, specifically, sea surface temperature, chlorophyll *a*, and fish age, consistent with an exposure gradient relationship. We verified that seasonal microbial changes were not confounded by regional migration of chub mackerel subpopulations by nanopore sequencing a 14,769-bp region of the 16,568-bp mitochondria across all temporal fish specimens. A cosmopolitan pathogen, Photobacterium damselae, was prevalent across multiple body sites all year but highest in the skin, GI, and digesta between June and September, when the ocean is warmest. The longitudinal fish microbiome study evaluates the extent to which the environment and host biology drives mucosal microbial ecology and establishes a baseline for long-term surveys linking environment stressors to mucosal health of wild marine fish.

**IMPORTANCE** Pacific chub mackerel, *Scomber japonicus*, are one of the largest and most economically important fisheries in the world. The fish is harvested for both human consumption and fish meal. Changing ocean conditions driven by anthropogenic stressors like climate change may negatively impact fisheries. One mechanism for this is through disease. As waters warm and chemistry changes, the microbial communities associated with fish may change. In this study, we performed a holistic analysis of all mucosal sites on the fish over a 1-year time series to explore seasonal variation and to understand the environmental drivers of the microbiome. Understanding seasonality in the fish microbiome is also applicable to aquaculture production for producers to better understand and predict when disease outbreaks may occur based on changing environmental conditions in the ocean.

## Abstract (precis)

Pacific chub mackerel, *Scomber japonicus*, are one of the largest and most economically important fisheries in the world. The fish is harvested for both human consumption and fish meal. Changing ocean conditions driven by anthropogenic stressors like climate change may negatively impact fisheries. One mechanism for this is through disease. As waters warm and chemistry changes, the microbial communities associated with fish may change. In this study, we performed a holistic analysis of all mucosal sites on the fish over a 1-year time series to explore seasonal variation and to understand the environmental drivers of the microbiome. Understanding seasonality in the fish microbiome is also applicable to aquaculture production for producers to better understand and predict when disease outbreaks may occur based on changing environmental conditions in the ocean.

## INTRODUCTION

Pacific chub mackerel, Scomber japonicus (Houtuyn 1782), is an economically and ecologically important, cosmopolitan, marine coastal pelagic fish found in the temperate and tropical waters of the Pacific, Atlantic, and Indian Oceans (1, 2). *S. japonicus* is currently the fifth largest commercial fishery (purse seine) in the world (3), processed for human consumption and animal feed. In the United States, *S. japonicus* was a prominent commercial fishery but has been on the decline since the 1980s due to a collapse in spawning and fishery stock biomass, leading to the last U.S. mackerel cannery closing in 1992 (4). The boom and bust cycles of the fishery have been attributed to large-scale environmental factors, including climate variability patterns such as the Pacific Decadal Oscillation and North Pacific Gyre Oscillation, as well as physiochemical and biotic variables, including sea surface temperature, sea level, upwelling, and chlorophyll *a* (5–8). Juveniles grow quickly, reaching 50% of total growth by the first 1.5 years of life, with larval growth highest in warmer water (16.8 to 22.1°C) (9). Larvae eat copepods and zooplankton (3), while juveniles and adults consume primarily small fish and pelagic crustaceans (2). *S. japonicus* is an important prey item for marine mammals, sea birds, and higher trophic-level fish, such as tunas and sharks (2). In the eastern North Pacific, *S. japonicus* migrates north in the summer and south in the winter (10), with seasonal offshore migrations occurring from March to May. Climate change and warming oceans likely have contributed to stocks shifting to more northerly migrations (4). Modeling has shown that nearly 90% of the *S. japonicus* catch was explained by temperature (28 to 29.4°C), salinity (33.6 to 34.2 PSU), and chlorophyll *a* (0.15 to 0.5 mg/m^3^) (8), whereas rates of survival of recruits to 1 year were highly associated with low plankton biomass (11). *S. japonicus* fish are ecologically and commercially important while occupying broad environmental gradients. This combined with their relative ease of collection make them an excellent model to study the environmental and biological drivers of microbiome diversity in a marine vertebrate.

The primary mucosal surfaces of fish include the gills, skin, and throughout the gastrointestinal tract (GI), all of which are important to fish health. Disease resistance in the host is promoted in the mucus through continual epithelial shedding and immune cell regulation (12, 13). The mucus is an important physical barrier to the environment and is generally thought to be colonized with a unique microbiome (14). The skin and gut both have mucosa-associated lymphoid tissues which produce IgT^+^ B cells protecting the host from invasion of mucosal microbiota (15). The establishment of microbiomes on mucosal sites is a function of exposure to environmental matrices (seawater, sediment, and food/prey items) and successful colonization. For example, the gills and skin are constantly bathed in seawater, while the GI experiences variable exposure to nutrients and foodborne microbial communities during feeding. Successful colonization within mucosal sites is further driven by variables regulated by the host, which can include different physiological conditions of the host, including the immune status. Various protective enzymes related to the innate immune response, including lysozymes, proteases, phosphatases, esterases, and sialic acid, can be differentially abundant in the mucus depending on the host fish exposure to environmental microbes (16).

To understand the full microbiome potential of a given host, it is important to evaluate the variability longitudinally throughout an entire season (year) and to continue sampling throughout consistent periods for multiple years. Such studies in fish species that are exposed to a dynamic and variable aquatic environment are lacking. Including long-term biological monitoring of commercially and ecologically important marine fish to complement the >100 years of seawater temperature and salinity data taken from the Scripps Institution of Oceanography (SIO) Pier will be important for understanding marine ecosystem dynamics. Although most ecological studies since 2004 span less than a year and have sampling frequencies of 1 month or greater (17), we have designed our study to include 38 sampling events across 1 year. Previous work investigating seasonal or temporal microbiome changes in the marine environment has focused on free-living pelagic seawater microbes (18). Examining seawater communities over a 6-year period, Gilbert et al. found that day length described over 65% of microbial community diversity, with richness highest during winter months in the North Atlantic (19). Very few time series data sets spanning an entire year exist for analyzing the host-associated microbiome, particularly for ecological species. Within humans, most seasonally active microbes in the gut are associated with populations spending more time outdoors, suggesting that seasonal variance in the environment has a greater influence on those with higher environmental exposure (20). In freshwater fish, lower microbial diversity and altered composition in the gut were associated with warmer summer months in tilapia reared in earthen ponds (21). In farmed salmon, however, no seasonal variations of gut microbiota composition were detected, although alpha diversity was highest during warm-water months (22).

The purpose of this study was to quantify the effects of environmental and biological drivers across unique mucosal body sites in a marine fish over a longitudinal time course spanning 1 year. From 28 January 2017 to 26 January 2018, 229 pacific chub mackerel, Scomber japonicus, were collected off the SIO Pier over 38 sampling events. Mucosal microbiome communities were sampled from five body sites, including gill, skin, digesta, GI, and pyloric ceca, within each fish. Seawater and marine sediment samples were also collected at points of landing to compare mucosal microbial communities to potential environmental sources. Microbiome processing was performed using the Earth Microbiome protocol using the 16S rRNA gene V4 region. Water conditions (salinity, temperature, pressure, and chlorophyll *a*) and fish biometrics (length, mass, condition factor, and age) were collected and compared to mucosal microbiomes to determine significant ecological drivers. We evaluated both alpha diversity measures (Shannon entropy and Faith’s phylogenetic diversity [PD]) and beta diversity (weighted and unweighted UniFrac) to assess these changes. In addition, we calculated microbial gamma diversity across body sites and time to understand effects of sampling effort on capturing true host microbiome diversity. Our results show that mucosal communities across body sites are highly differentiated in a single species of marine fish and that seasonal environmental drivers partially account for this differentiation.

## RESULTS

### Microbial diversity associated with a marine pelagic fish across body sites over 1 year.

**FIG 1:**
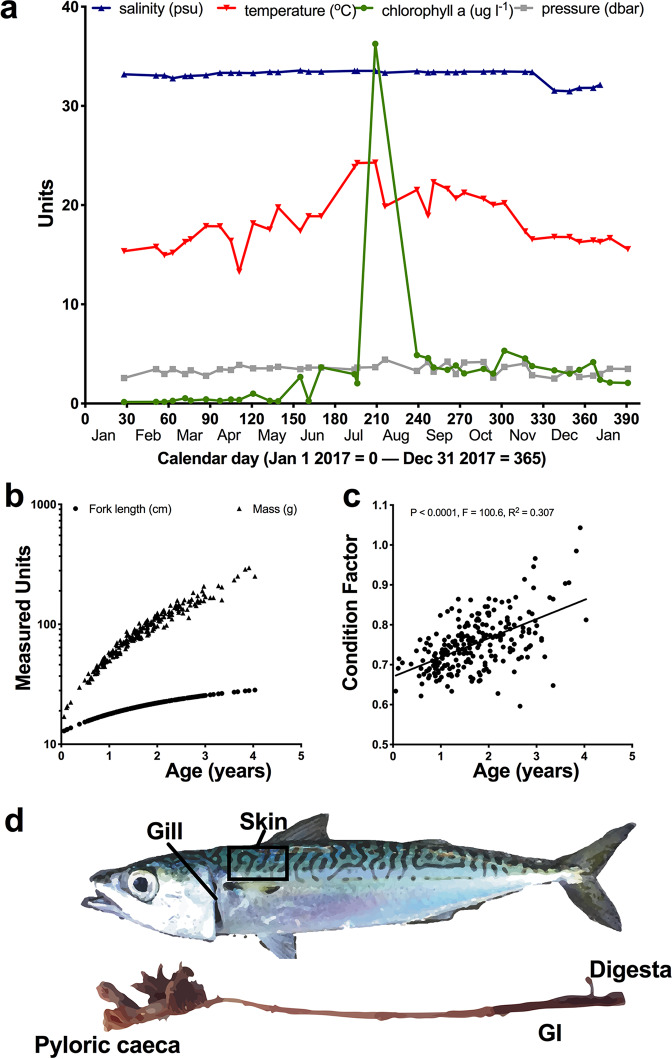
Environmental sampling design. Throughout 2017 and into early 2018, 229 Pacific chub mackerel, *S. japonicus*, were caught across 38 sampling events from the SIO Pier. (a) Pier seawater measurements of temperature, salinity, pressure, and chlorophyll *a* were collected using the scoos.org database. (b) Ages of *S. japonicus* were inferred from fish lengths. (c) Condition factor was calculated for each fish based on length and mass. (d) Mucosal microbiome samples were collected from five body sites, including gill, skin, pyloric ceca, GI, and fecal or digesta material removed from the lower GI.

From January 2017 to January 2018, 229 wild *Scomber japonicus* mackerel were collected from the SIO Pier across 38 sampling events at approximately 5 fish per week, although actual takes varied due to weather and other constraints. Seawater temperature, salinity, pressure, and chlorophyll *a* were recorded using the SCOOS online database (Fig. 1a). Fork length and mass were recorded and approximate age of the fish was determined from length (Fig. 1b). The condition factor of the fish was positively associated with older fish (*P* < 0.0001; *R*^2^ = 0.307) (Fig. 1c). Along with paired seawater samples, mucosal microbiome samples were sequenced from the gill, skin, digesta, GI, and pyloric ceca of each fish (Fig. 1d).

**FIG 2:**
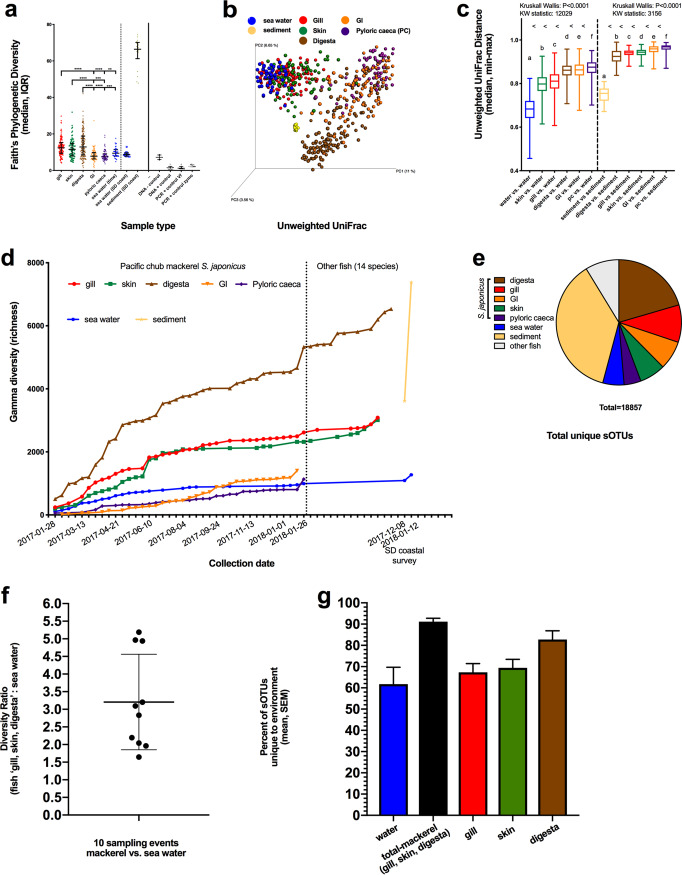
Microbial diversity of coastal environmental controls and *S. japonicus* mucosal microbiome. (a) Alpha diversity was calculated using Faith’s phylogenetic diversity metric in Qiime2, with the median and interquartile range displayed. (b) Principal-coordinate analysis (PcoA) plot of beta diversity as calculated using unweighted UniFrac with a rooted phylogenetic tree inserted using the SEPP method in Qiita and Qiime2. (c) Distances of mucosal microbial communities (gill, skin, digesta, GI, and pyloric ceca) compared to seawater and sediment samples using nonparametric Kruskal-Wallis test. (d) Accumulation of total microbial diversity across chronological sampling events within fish (*S. japonicus* and 14 other species) mucosal sites, water samples, and sediment. (e) Proportion of unique microbial diversity (sOTUs) contributed by body site or environment to the whole data set. (f) Comparison of 1 volume of fish to 1 volume of seawater: ratio of cumulative microbial richness from fish mucus (gill, skin, and digesta) to seawater from 10 unique mackerel across 10 sampling events. (g) Uniqueness of an environment as described by average contribution of unique sOTUs to the marine ecosystem: percentage of sOTUs in a given environment which are unique to that environment and not shared in other sample types.

A total of 612 samples resulting in 18,857 sub-operational taxonomic units (sOTUs), processed with the miniaturized PCR method, passed the sample exclusion criteria. To determine which samples had detectable levels of microbial material, and thus exclude the failures (i.e., the minimal read count for sample exclusion criteria), we applied the KatharoSeq method. The read counts from DNA extraction-positive controls of various cell counts were compared to compositional readout, and the read count at which 90% of the reads mapped appropriately was chosen as the rarefaction depth, which was 1,362 reads (see Fig. S1 in the supplemental material). Further details of the method can be found in the KatharoSeq paper (23). Alpha diversity measured by Faith’s PD was significantly different compared across mackerel body sites and seawater (Kruskal-Wallis, *P* < 0.0001; KW (Kruskal-Wallis) statistic, 87.48) (Fig. 2a). Gill, skin, and digesta samples had higher diversity than the GI and pyloric cecum samples, while gill and digesta had higher diversity than seawater (Fig. 2a). Beta diversity indicates that the gill and skin mucosal samples clustered more closely to seawater than digesta, GI, and pyloric ceca, which also had higher within-body-site variability (Fig. 2b). Some of the digesta samples also appeared to cluster more closely to sediment samples. When tested, skin samples followed by gill samples were found to be most similar to seawater samples, whereas the digesta samples were most similar to sediment (Fig. 2c). To understand sample size requirements for capturing novel microbial diversity associated with fish, we compared accumulation of microbial richness over the 1-year sampling period across all sample types. Overall microbial richness in the gill, skin, GI, pyloric ceca, and seawater appeared to level off after only a couple of months (20 to 50 samples), whereas digesta samples continued to increase, perhaps requiring another few years of data collection to approach saturation. For comparison, we included gill, skin, and digesta samples from 14 other local San Diego species of fish (right of the dotted line in Fig. 2d). Digesta diversity increased with the addition of the first new species and followed a similar trend, while gill and skin samples did not increase much, suggesting an overall conservation of microbes in other species of fish. Lastly, total gamma diversity, or richness, was calculated for all samples in this study; the results showed that sediment samples had the most microbial diversity, followed by mackerel digesta and mackerel gill (Fig. 2d). When comparing all samples, it was found that the total unique microbial diversity in a single species of fish, *S. japonicus*, was 8.8-fold greater than that in seawater (9,172 versus 1,039 sOTUs) (Fig. 2e). One may, then, consider this question: how much microbial diversity is in 1 unit of fish compared to 1 unit of seawater? To determine this ratio on a per-sample basis, we compared 10 unique fish sampled each from 10 different sampling events spanning the 1-year study. On average, the cumulative fish mucosal microbiome of the top body sites (gill, skin, and digesta) had 3.2 times (range, 1.7 to 5.2) more microbial diversity than a comparative unit of seawater (Fig. 2f). In addition, we determined the number of sOTUs unique to each of these four environments (Fig. S2). In seawater, an average of 61.8% of the sOTUs were found only in seawater, whereas an average of 91.1% of cumulative fish microbes were associated only with fish (Fig. 2g), demonstrating the potential for microbial discovery within and upon fish hosts in the ocean.

10.1128/mSphere.00401-20.1 FIG S1 Limit-of-detection titration curves generated from controls to determine sample exclusion criteria of 1,362 reads. Download FIG S1, PDF file, 0.04 MB. Copyright © 2020 Minich et al. 2020 Minich et al. This content is distributed under the terms of the Creative Commons Attribution 4.0 International license.

10.1128/mSphere.00401-20.2 FIG S2 Representation of shared sOTUs between major fish mucosal body sites (gill, skin, and digesta) versus seawater samples taken from 10 unique fish across 10 subsequent sampling events spanning 1 year. “S#” refers to the sampling event number (s1 to s38). Download FIG S2, PDF file, 0.4 MB. Copyright © 2020 Minich et al. 2020 Minich et al. This content is distributed under the terms of the Creative Commons Attribution 4.0 International license.

### Environmental and biological drivers of the *S. japonicus* mucosal community.

We next quantified the combined and specific effects of four environmental variables, including chlorophyll *a* concentration, sea surface temperature, salinity, and pressure, along with four biological variables, including fish age, fork length, mass, and condition factor, on the fish-associated mucosal microbiomes. Alpha diversity measures were assessed using the general linear model (GLM). For alpha diversity measures of Shannon diversity, skin mucus was significantly influenced by the factors (*P* < 0.001; *R*^2^ = 0.38; F-statistic [F-stat], 6.595), with chlorophyll *a* having a negative association and temperature a positive association (*P* < 0.0001; *P* = 0.0004). Gill samples were not assessed because the Shannon diversity did not meet the assumptions of normally distributed residuals (Shapiro test, *P* < 0.05) and was not homoscedastic (Breusch-Pagan, *P* < 0.05) (Table 1). For the alpha diversity measure of Faith’s phylogenetic diversity (PD), which takes into account phylogenetic diversity with richness, all data were log transformed to meet the assumptions of the GLM. The gastrointestinal tract samples, however, still did not meet the assumptions, as the residuals were not normally distributed (Shapiro test, *P* < 0.05) and thus were excluded from analysis. Gill, skin, and pyloric cecum Faith’s PD were significantly influenced by the measured factors (gill, *P* < 0.0001, *R*^2^ = 0.33, and F-stat = 7.042; skin, *P* = 0.00039, *R*^2^ = 0.26, and F-stat = 4.239; and pyloric ceca, *P* = 0.00891, *R*^2^ = 0.22, and F-stat = 2.972). The gill sample diversity was negatively associated with chlorophyll *a* concentration (*P* = 0.00549). Skin was negatively associated with chlorophyll *a* concentration (*P* = 0.00182) and age (*P* = 0.00811) while positively associated with temperature (*P* = 0.04434). The pyloric cecum was positively associated with age (*P* = 0.04787) and temperature (*P* = 0.00305) while negatively associated with salinity (*P* = 0.04921).

**TABLE 1:** Quantification of environmental and biological variables on fish mucosal microbiomes as measured by alpha diversity with generalized linear model^*a*^

Body site	Alpha diversity	Adj. *R*^2^	F-stat	*P* value	*P* value							
					Host (biometrics)				Environmental (water conditions)			
					Age (yrs)	FL (mm)	Mass (kg)	K NA	Chl *a* (μg liter^−1^)	Press (Dbar)	Sal (PSU)	Temp (°C)
Gill^*b*^	Shan	0.13	2.89	0.006	—^*d*^	—	—	—	—	—	—	—
Skin	Shan	0.38	6.60	<0.0001	—	—	—	—	<0.001 (−)	—	—	<0.001 (+)
Digesta	Shan	−0.01	0.84	0.567	—	—	—	—	—	—	—	—
GI	Shan	−0.01	0.94	0.494	—	—	—	—	—	—	—	—
PC	Shan	0.05	1.35	0.244	0.027 (+)	0.026 (+)	—	—	—	—	—	—
Gill	PD	0.33	7.04	<0.0001	—	—	—	—	0.005 (−)	—	—	—
Skin	PD	0.26	4.24	<0.0001	0.008 (−)	—	—	—	0.002 (−)			0.044 (+)
Digesta	PD	0.02	1.29	0.258	—	—	—	—	—	0.036 (−)	—	—
GI^*c*^	PD	−0.01	0.91	0.514	—	—	—	—	—	—	—	—
PC	PD	0.22	2.97	0.009	0.048 (+)	—	—	—	—	—	0.049 (−)	0.003 (+)

a For *P* values, “(−)” indicates a negative association and (+) indicates a positive association. Shan, Shannon diversity; PD, Faith’s phylogenetic diversity (log transformed); FL, fork length; K, Fulton’s condition factor; Press, pressure; Sal, salinity; PC, pyloric ceca.

b Shannon diversity of gill sample results were excluded from analysis because residuals are nonnormal and because of homoscedasticity.

c Faith’s phylogenetic diversity for GI sample results were excluded from analysis because residuals are nonnormal. Shapiro, residual normality > 0.05; Breusch-Pagan, homoscedasticity > 0.05.

d —, not significant.

The extent to which environmental and biological variables explain microbial diversity was also assessed for beta diversity, including both unweighted UniFrac and weighted UniFrac distances. Adonis permutational multivariate statistical analysis was used to test overall significance along with variance explanation by factor. Unweighted UniFrac distance measures showed that gill, skin, and digesta samples were influenced by measured factors (Adonis, *P* < 0.0001, *R*^2^ = 0.12, *R*^2^ = 0.15, and *R*^2^ = 0.09). The gill was primarily driven by chlorophyll *a* concentration and age, while skin was influenced mostly by chlorophyll *a*, age, and fork length. For weighted UniFrac distances, both gill (*P* < 0.0001 and *R*^2^ = 0.14) and skin (*P* = 0.001 and *R*^2^ = 0.20) were significantly influenced by factors, with age being the most significant driver. In summary, the skin mucosal microbiome was significantly influenced by environmental and biological factors in each of the four measures across alpha (Shannon and Faith’s PD) and beta (unweighted and weighted UniFrac) diversity, while gill was significant in three measures (Faith PD, unweighted UniFrac, and weighted UniFrac). The environmental variables of chlorophyll *a* followed by temperature had the most frequent influences on microbial communities across body sites, while age was the most frequent biological factor (Table 2). For alpha diversity measures, temperature was positively associated with Shannon diversity in skin, along with Faith’s PD in skin and pyloric ceca (*P* < 0.05). Chlorophyll *a* was negatively associated with Shannon diversity in skin, along with Faith’s PD in gill and skin (*P* < 0.05). Age was negatively associated with Faith’s PD in skin and positively associated with Faith’s PD in pyloric ceca (*P* < 0.05). Salinity was negatively associated with Faith’s PD in pyloric ceca (Table 1). For beta diversity measures, age was associated with unweighted and weighted UniFrac distances in the gill and skin, while chlorophyll *a* was associated with unweighted UniFrac in gill and skin.

**TABLE 2:** Quantification of environmental and biological variables on fish mucosal microbiomes as measured by beta diversity with multivariate statistics (Adonis)

Body site	Beta diversity^*a*^	Total *R*^2^	*P* value	*P* value							
				Age (yrs)	FL (mm)	Mass (kg)	K NA	Chl *a* (μg liter^−1^)	Press (Dbar)	Sal (PSU)	Temp (°C)
Gill	u UniF	0.12	<0.0001	<0.001	—^*b*^	—	—	<0.001	—	—	—
Skin	u UniF	0.15	<0.0001	<0.001	<0.001	—	—	<0.001	—	—	—
Digesta	u UniF	0.09	<0.0001	—	—	—	—	—	—	—	—
GI	u UniF	0.14	0.099	—	—	—	—	—	—	—	—
PC	u UniF	0.15	0.32	—	—	—	—	—	—	—	—
Gill	w UniF	0.14	<0.0001	<0.001	—	—	—	—	—	—	—
Skin	w UniF	0.20	0.001	<0.001	—	—	—	—	—	—	—
Digesta	w UniF	0.10	0.038	—	—	—	—	—	—	—	—
GI	w UniF	0.13	0.38	—	—	—	—	—	—	—	—
PC	w UniF	0.14	0.49	—	—	—	—	—	—	—	—

a u UniF, unweighted UniFrac; w UniF, weighted UniFrac.

b —, not significant.

### Population structure of *S. japonicus*.

*S. japonicus* fish are thought to have three spawning populations along the Pacific coast of North America (4), which suggest that our environmental and biological associations could be explained in part by population dynamics over the year. To estimate the changes in *S. japonicus* population structure over our study, we sequenced two fragments of mitochondrial DNA directly from skin mucus genomic DNA (gDNA) for a total length of 14,769 bp for 93 fish samples landed between 27 August 2017 and 26 January 2018 to span the temperature extremes (summer and winter). Two samples were removed from the analysis due to having lower coverage (less than 10×) or more than 20 N’s in the consensus sequence (Fig. S4a). The majority of samples (93%) had at least 100× coverage of the mitochondria target region (Fig. S4b). Based on near full-length mitochondrial data, no population structure was observed, consistent with our sampling of one population of *S. japonicus* over the course of the study (Fig. S5).

10.1128/mSphere.00401-20.3 FIG S3 Core analysis plot describing the total number of sOTUs (a) and total fraction of sOTUs (b) found in 10%, 20%, 30%, 40%, 50%, 60%, 70%, 80%, 90%, and 100% of samples. Shaded area highlights microbes found in at least 30% of samples from a given body site or sample type. Download FIG S3, PDF file, 0.04 MB. Copyright © 2020 Minich et al. 2020 Minich et al. This content is distributed under the terms of the Creative Commons Attribution 4.0 International license.

10.1128/mSphere.00401-20.4 FIG S4 Long-read sequencing of mitochondria amplicon fragments 1 and 2. (a) Total coverage per base position across both fragments across 92 samples with number of N’s reported. (b) Summary statistic on coverage bins per samples. Download FIG S4, PDF file, 0.7 MB. Copyright © 2020 Minich et al. 2020 Minich et al. This content is distributed under the terms of the Creative Commons Attribution 4.0 International license.

10.1128/mSphere.00401-20.5 FIG S5 Phylogenetic tree of near-full-length (14,769 bp) mitochondrial DNA sequences across 91 *S. japonicus* fish sampled between 27 August 2017 and 26 January 2018. Outgroups include two other *Scomber* species: *S. colias* and *S. australasicus*. Download FIG S5, PDF file, 0.2 MB. Copyright © 2020 Minich et al. 2020 Minich et al. This content is distributed under the terms of the Creative Commons Attribution 4.0 International license.

### Microbial associations with body sites.

**FIG 3:**
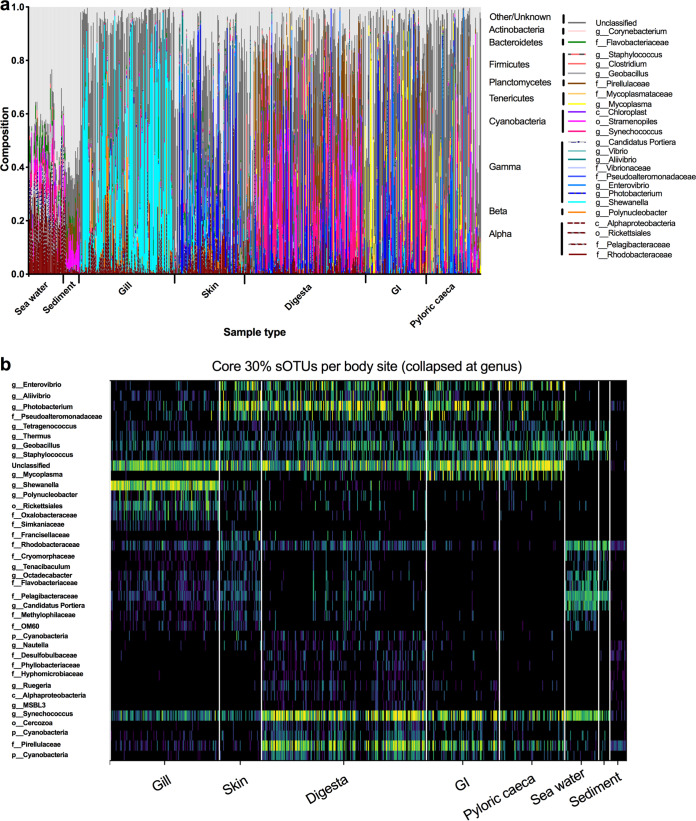
Microbial summary across fish body sites and environment controls. (a) Top 25 most abundant genera across body sites. (b) Core microbes: all sOTUs found in at least 30% of samples from a given sample type (gill, skin, digesta, GI, pyloric ceca, and seawater) collapsed at the genus level (or lowest common ancestry) and overall clustered by similar distribution on the heat map.

Microbial diversity was unique within the various *S. japonicus* body sites and environment (seawater and sediment), with the top 25 most abundant genera making up the majority of reads (Fig. 3a). We performed a core analysis to determine which groups of sOTUs were most prevalent across various body sites. Specifically, sOTUs present in at least 30% of samples from a given sample type were indicated as the core (Fig. 3b and Fig. S2). The most common fish-associated microbial group was “unclassified.” This group was made up of 12 different sOTUs which were most similar by BLAST (99 to 100% query coverage; 78 to 80% sequence identity [ID]) with the rRNA of *Eimeria* within the Apicomplexa phylum. When summed together, these core Apicomplexa sOTUs were detected in 97% of gill and pyloric cecum samples, 91% of GI samples, 87% of digesta samples, and 61% of skin samples and made up the greatest proportion of reads in the pyloric cecum samples compared to other sample types (median relative abundances, gill, 0.023; skin, 0.001; digesta, 0.006; GI, 0.014; and pyloric ceca, 0.17). Furthermore, this Apicomplexa sOTU group was not found in any of the sediment samples and was found in only 1 out of the 50 water samples. In comparing other Southern California reef fish collected in this study, these sOTUs were not found in the gill or skin samples from other Southern California reef fish and were found only in 2 out of 21 digesta samples (in very low proportions) from the other reef fish, suggesting a high probability for the uniqueness to the *Scomber japonicus* host. *Synechococcus* was the most cosmopolitan bacterial genus found in all sample types, especially seawater, digesta, GI, pyloric ceca, and skin. *Rhodobacteraceae* were found in all environments, particularly seawater, with lower relative abundances on the gill, skin, digesta, and GI body sites. Other seawater-associated taxa (family description) at the lowest common ancestor level included *Cryomorphaceae*, *Tenacibaculum* (*Flavobacteriaceae*), *Octadecabacter* (*Rhodobacteraceae*), *Pelagibacteraceae*, *Candidatus* Portiera, and *Methylophilaceae*. The gill was dominated by three sOTUs within the genus *Shewanella* (*Shewanellaceae*), along with six sOTUs from the order *Rickettsiales* and one from the genus *Polynucleobacter* (*Burkholderiaceae*). There were 10 sOTUs which were unable to be classified taxonomically. Seawater microbes, including *Rhodobacter* (*Rhodobacteraceae*) and *Pelagibacter* (*Pelagibacteraceae*), were also present on the gill in lower numbers. The skin community was dominated by various sOTUs from the *Vibrionales* order, including *Photobacterium* and *Enterovibrio*. Other general water-associated microbes, such as *Rhodobacteraceae*, *Synechococcus* (*Synechococcaceae*), *Flavobacteriaceae*, and *Pelagibacteraceae*, were also part of the core but made up a smaller fraction of the microbial community. Within the digesta, GI, and pyloric ceca, all of the *Vibrionales*-associated sOTUs, namely, *Enterovibrio* and *Photobacterium*, were part of the core and also had high relative abundances in the GI. These microbes are of interest due to their potential pathogenicity. Digesta samples were comprised of many seawater-dwelling Cyanobacteria, including *Synecochoccus* (*Synechococcaceae*), but were also high in the family *Pirellulaceae* and sporadically large amounts of the genus *Clostridium* (*Clostridiaceae*). *Mycoplasma* (phylum *Tenericutes*) was a dominant genus in the GI and pyloric ceca.

### Candidate pathogen and probiotic associations.

**FIG 4:**
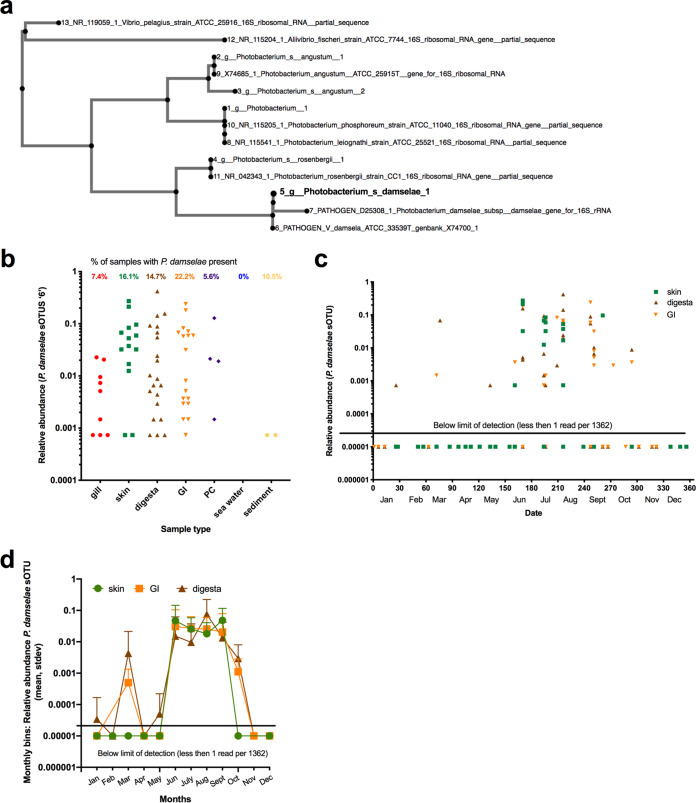
Prevalence of marine vertebrate pathogen Photobacterium damselae on *S. japonicus* body sites throughout the sampling effort. (a) Validation of P. damselae 148-bp v4 region by way of phylogenetic comparison to two known pathogenic isolates and nonpathogenic strains. (b) Total relative abundance of P. damselae sOTU across five body sites and environments for successfully sequenced samples. Total prevalence or percentage of samples with *P. damelsae* present is also calculated for each sample type and displayed at the top of graph (7.4% gill samples, 16.1% skin, 14.7% digesta, 22.2% GI, 5.6% pyloric ceca, 0% water, and 10.5% sediment). (c and d) Proportion of microbial community comprised of P. damselae sOTU across the most prevalent body sites (skin, digesta, and GI) over the 38 sampling events (c) or binned by month across 1 year (d). Relative abundance is calculated as number of P. damselae sOTU reads divided by 1,362, the rarefaction number. Any samples with 0 P. damselae reads are considered under the detection limit and are displayed as equal to 0.00001 relative abundance in order to visualize on the log scale.

Various *Bacillus* spp. and *Lactobacillus* spp. were found to be present across multiple body sites (Fig. S6). As expected, seawater contained many common groups, including *Synechococcus*, *Rhodobacteraceae*, *Pelagibacter*, and *Flavobacteriaceae*, while the sediment had consistently higher levels of *Pirellulaceae* (Fig. 4).

10.1128/mSphere.00401-20.6 FIG S6 Prevalence of candidate probiotics *Bacillus* spp. and *Lactobacillus* spp. on *S. japonicus* body sites throughout the sampling effort. Shown are proportions of the microbial community comprised of *Bacillus* and *Lactobacillus* sOTUs across the body sites and environment over the sampling effort of 1 year. Relative abundance is calculated as number of sOTU reads divided by 1,362, the rarefaction number. Any samples with 0 *Bacillus* or *Lactobacillus* reads are considered under the detection limit and are not displayed. Download FIG S6, PDF file, 0.04 MB. Copyright © 2020 Minich et al. 2020 Minich et al. This content is distributed under the terms of the Creative Commons Attribution 4.0 International license.

Among the *Vibrionales*, there were five highly abundant *Photobacterium* sOTUs present in the skin and gastrointestinal tract, which prompted further phylogenetic evaluation to elucidate species-level assignments. The full 16S rRNA genes of two pathogenic isolates of Photobacterium damselae, four other *Photobacterium* species, including Photobacterium angustum, Photobacterium phosphoreum, Photobacterium leiognathi, and Photobacterium rosenbergii, and two *Vibrio* species as outgroups were aligned with the five *Photobacterium* unique sOTUs. The phylogenetic tree (Fig. 4a) of the five *Photobacterium* sOTUs in this data set with the known *Photobacterium* strains is able to identify and resolved the taxonomic assignments. The Photobacterium damselae sOTU identified in our data set was 100% identical to the v4 region of 16S rRNA from the two pathogenic strains while distinct from the other *Photobacterium* species. This P. damselae sOTU was identified across various body sites of fish, but was most prevalent in the GI, skin, and digesta samples (present across 22.2%, 16.1%, and 14.7% of samples, respectively) (Fig. 4b). For the GI, skin, and digesta samples which had P. damselae present, the single P. damselae sOTU made up 5.88%, 6.99%, and 5.32% of the total microbial composition, respectively. Further, the temporal enrichment and prevalence of this P. damselae sOTU were highest between June and September, coincident with the highest sea surface temperatures sampled.

## DISCUSSION

Our study evaluated how the mucosal microbial community of a wild marine fish species is influenced according to environmental and biological variance as experienced over the course of an annual season in coastal temperate waters. Body sites had unique microbial signatures that were differentially influenced by environmental and biological measures. Cumulatively, the mackerel microbiome has, on average, 3.2 times more microbial diversity than a similar volume of seawater, with 91.1% of the microbes found only in association with fish mucosal surfaces, demonstrating the vast microbial bioprospecting potential of marine fish. We further demonstrate the need to sample large numbers of fish replicates (*n* = 43 to 84 individuals), depending on body site, to identify 90% of the resident microbial community. Alpha diversity was higher in the gill, skin, and digesta communities than in the gastrointestinal tract and pyloric ceca. Beta diversity measures demonstrated that fish mucosal sites were primarily driven by body site location and were unique compared to the surrounding environment. An exposure gradient was observed, with skin and gill surfaces being more similar to the water column while the digesta community was more similar to sediments. Further, the environmental and biological variables best explained variation in the skin and gill microbiomes as opposed to the internal body sites (digesta, GI, and pyloric ceca). A novel Apicomplexa, parasitic alveolate specific to the *Scomber japonicus* fish species was discovered which had a high prevalence in all body sites, particularly the gill and pyloric ceca. Relatively little is known about the genetics and evolution of Apicomplexa parasites, but several coccidia, including *Eimeria*, *Goussia*, and *Calyptospora*, have been recently identified in various species of commercially important marine fish (24). Lastly, an important fish pathogen, Photobacterium damselae, was observed in high prevalence on GI, skin, and digesta communities and was associated with the summer months, which exhibit higher temperatures and low nutrients.

Regardless of environmental conditions, the mackerel mucosal body site was the strongest driver of microbiome diversification, with each site associated with a specific gradient of environmental exposure. The gill and skin communities were most similar to the seawater, whereas the gastrointestinal samples were more divergent. This environmental gradient, which distinguishes host-associated gut microbes from free-living microbes, was first described for mammals (25). While environmental exposure gradients have also been shown to influence gut or skin microbiomes in amphibians, fish (26, 27), and other vertebrates (28, 29), the current study provided a community assessment that explicitly tested this comparison across multiple mucosal surfaces in fish. Marine fish differ from other vertebrates in that their microbial exposure rates are greatly elevated compared to those of terrestrial or freshwater species. Seawater can harbor 1 million cells per ml (30), while coastal sediments can be 2 orders of magnitude higher, at 100 million cells per cm^3^ (31). Gill microbial communities may be supported physically by the complex anatomical structures of laminae and filaments and chemically through gas exchange, ion transport, and waste excretion. Age, phylogeny, and diet have been implicated as influencing the gill microbial community in tropical fish, with *Shewanella* taxa being dominant (32). The skin microbiomes of tropical marine fish have been also shown to be driven by phylogeny and diet (33). Few studies, however, have evaluated these body sites in temperate marine fish. Digesta and GI samples in *S. japonicus* were the most variable, suggesting that either niche differentiation is more static in the gill and skin environments or microbial turnover is lower. Another, more likely explanation is that the high variability of the mackerel diet (omnivores) and feeding frequency would contribute to a higher gut microbiome variability, which has been seen with humans and plant consumption (34). Although not assessed in this study, total stomach content mass may vary up to 3-fold and differ compositionally (ranging from zooplankton to euphausiids) between spring and autumn in Atlantic mackerel (35). Diet also changes with ontogeny in many fishes, including *Scomber japonicus*, as prey acquisition is limited by skull and mouth morphology, including jaw length and degree of mouth opening (36). The discovery of novel microbial lineages and metabolic activity should focus on fish mucosa-associated environments, specifically the gill, skin, and digesta communities, which had the highest levels of phylogenetic diversity in our data set. Sediment samples had the highest diversity, yet were most similar, thus having the lowest intersample variability.

Environmental and biological variables best explained the external (skin and gill) microbiomes compared to the internal GI communities. Among the environmental variables, chlorophyll *a* concentration followed by temperature and salinity was the strongest driver, while age was the most pronounced of the biological metrics. Chlorophyll *a* concentration is a general indicator of primary production and microbial growth or proliferation in the water column. As phytoplankton blooms occur in the ocean due to nutrient enrichments through upwelling or eutrophication, bacterial communities in the water column also change, thus altering exposure to fish and other marine animals. While many studies have examined the effects of harmful algal blooms on marine organisms (37), few have quantified the extent of these exposures in the wild. Temperature has been shown to influence marine macroalgae (38) and oyster hemolymph microbiomes (39). Salinity was one of the first major abiotic conditions shown to drive microbiome differentiation in free-living freshwater versus marine environments (40) and has also been shown to influence fish microbiomes deterministically (41). Fish gill parasite load has been shown to be positively associated with fish age, season, eutrophic water conditions (42), and temperature (43). This may be explained by increased biofouling activity or biofilm formation over time on the gills or could be a response to parasite persistence. Unfortunately, we did not measure parasite abundances on the gill, but this would be an important area of research to examine the impact of parasite load on microbiome diversity and vice versa. Understanding the effects of age on the microbiome was first demonstrated in African turquoise killifish, in which it was shown that microbiomes from older fish were associated with inflammation in the gut which could be rescued by fecal microbiome transplants from younger fish (44). It has been suggested that during host aging, gut communities of vertebrates may shift from commensal to pathogenic, leading to increased inflammation and overall dysbiosis (45). Our results indicate that microbial communities from other body sites may also be influenced by aging or development of fish and are deserving of additional research. Additional host-associated explanatory variables not measured in our study include diet, trophic level, and host genotype. However, our assessment did determine, based on mitochondrial DNA, that the genetic population of mackerel sampled was homogeneous, which further emphasizes the importance of environment and fish development stage on driving microbial community structure. Mitochondrial markers have been used for decades for resolving phylogenetic relationships between animals at the species level and more recently to identify subpopulations (46). Our study is the first to use nanopore sequencing, a 4th-generation sequencing platform, to multiplex (sequence multiple individuals at once) mitogenomes. The benefit of using a long-read sequencing technology is that the full length of the mitochondria can be sequenced in a single sequence run and may eventually be used to identify individuals from environmental DNA samples. Short-read methods require a subset of marker genes or loci to be used to identify population structure. Nanopore sequencing is a less expensive alternative to other single-molecule or synthetic long-read sequencing technologies, such as PacBio, 10× Genomics, or Illumina TSLR, and its utility in ecological genomic applications are beginning to be established.

Along with being most influenced by environmental and biological factors, overall the skin and gill communities were more similar phylogenetically to the seawater. Interestingly, of all mucosal sites, digesta samples were most similar to the sediment. The first explanation could be that mackerel feces seed the sediment. A second explanation is that the fish were feeding on benthic organisms (47), such as crustaceans buried in the sand. Although not quantified, we did find various types of crustaceans in the stomachs of the larger fish, along with occasional gritty material which appeared to be sediment (48). It is also possible that wave turbulence in nearshore environments where the fish were caught could also cause fish to be exposed to higher sediment levels through resuspension (49). Since sediments are often repositories of decaying organic matter, including anthropogenic contaminants, it is important to consider the negative health implications on a fish population as well as the potential human impacts associated with recreational fishing that occurs in nearshore locations, such as piers, and consumption of these fish. Understanding the natural source inoculum for the gastrointestinal tracts of wild fish may be important for conservation measures. If feeding ecology is deterministic of microbial exposure and subsequent colonization of either beneficial or detrimental function to the host, it would be important to understand how perturbations or changes to the environment modulate this process. Since the mucosal microbiomes of fish are more diverse than surrounding seawater, it is important to understand how migration patterns of fish may contribute to global marine microbial ecology and biogeochemistry. Wild birds, for instance, have been shown responsible for the transport and distribution of multidrug-resistant Escherichia coli (50), but such vector effects have not been investigated in fish.

Various potential pathogenic and beneficial microbes were persistently abundant across seasons, which has important implications for climate change and aquaculture. Various sOTUs within the order *Vibrionales*, including *Enterovibrio* and *Photobacterium*, were prevalent in over 30% of the skin and gut communities from fish samples. A putative Photobacterium damselae organism was present in skin and gut communities and in high relative abundance compared to other microbes. High abundance and prevalence across fish replicates could have important health implications, as this can be an important globally distributed fish pathogen (51) causing bacterial septicemia. On the other hand, the other major clade of *Photobacterium* species, including Photobacterium kishitanii, Photobacterium leiognathi, and Photobacterium mandapamensis, are important microbes for symbioses, as they often produce light, which some fish use to attract prey (52). While our analyses based on the 16S suggest that the sOTUs found on the mackerel were P. damselae, we cannot confidently say that they were pathogens, as we did not sequence or quantify the toxin genes. Furthermore, if P. damselae is an important host-associated microbe, understanding the conditions by which it becomes pathogenic will be important in modeling fishery impacts. As a pathogen, P. damselae has caused financial losses in marine fish farms across numerous species, including yellowtail, gilthead seabream, and seabass (53, 54), and is thought to be transferred through water (55) to other species, including humans (56). Chub mackerel are an important forage fish consumed by many higher-trophic-level fish, including tunas, billfish, and jacks, which could have implications for trophic transfer of pathogens warranting future studies. Further, this microbe was most prevalent and abundant during the summer months, suggesting that it could be associated with high water temperatures and low nutrients. Extending this time series for another 3 to 10 years will be crucial to continue monitoring population dynamics of this marine pathogen. While time series data sets exist for marine free-living microbial communities, few exist for marine vertebrates. Evaluating the extent by which exposure to marine pathogens influences disease is important for estimating impacts to fisheries. Further, as marine aquaculture activities continue to expand in coastal waters, farm monitoring of the host microbiome could be an important tool for preventing disease outbreaks and economic losses. Experimental mesocosm studies could also be useful to model this marine vertebrate pathogen. Examples in other vertebrates of widespread prevalence of opportunistic pathogens include 20 to 80% carriage rates of Staphylococcus aureus in humans (57).

Some novel candidate symbiotic interactions were discovered when evaluating microbial ecology across the various mucosal sites. In the gills, *Shewanella* spp. were highly prevalent, which is consistent with tropical fish microbiome studies (32) suggesting a potential symbiotic role. Some *Shewanella* organisms are common marine species responsible for eicosapentaenoic acid (20:5n-3, an omega-3 polyunsaturated fatty acid) production (58, 59) and have been documented for freshwater fish (60). Both *Bacillus* and *Lactobacillus* strains make up close to 50% of the microbial taxa in commercially available probiotics for aquaculture (61, 62). Therefore, mucus from wild marine fish could provide a novel source of probiotics for use in the aquaculture industry.

In our study, we have evaluated the full mucosal microbiome of a marine fish species over a 1-year period from a fixed location. The Pacific chub mackerel microbiome was primarily differentiated by mucosal body site. Environmental conditions and host biology primarily drive the skin and gill mucosal microbiomes, with chlorophyll *a* concentration, age, and temperature having the broadest effects. Our results provide the foundation to understanding natural microbiome variation in an ecologically and economically important wild marine fish and provide a basis for investigating how climate change and rising global sea surface temperatures may impact the marine fish microbiome and the biology of marine fishes.

## MATERIALS AND METHODS

### Sample collection *S. japonicus* time series.

From 28 January 2017 to 26 January 2018, 1 to 8 *Scomber japonicus* specimens were collected across 38 sampling events from the end of the SIO Pier (32.867, −117.257). Sea surface water samples were collected from each sampling event and immediately stored on dry ice. Environmental conditions at the time of sampling, including seawater temperature, salinity, pressure, and chlorophyll *a* concentration, were collected using publicly available data from the Southern California Coastal Ocean Observing System (SCCOOS) SIO Pier shore station data archive (http://www.sccoos.org) (Fig. 1a). Fishing occurred at or near sunset, with exact times recorded in the metadata (see Qiita Study ID 11721 for full metadata). Fish were caught using hook and line with a Sabiki rig, immediately euthanized upon landing using accepted protocols according to American Veterinary Medical Association (AVMA) guidelines, and stored on dry ice. Individual fish were wrapped in aluminum foil and handled with gloves prior to storage on dry ice to minimize contamination and then stored at –80°C for up to 6 months prior to dissection. During each fish sampling period, a 50-ml conical vial of surface seawater was collected from the SIO Pier using a winch. The water was immediately frozen on dry ice and stored in the same manner as the fish. Upon processing, frozen fish were weighed and measured, along with calculation of Fulton’s condition factor, which is a proxy for fish health (63, 64). Age was estimated using fish length as derived from the most recent Pacific chub mackerel stock assessment (4) (Fig. 1b and c) (65), where otoliths were compared to 25 fish individuals per catch across multiple years (1962 to 2008). Specifically, the von Bertalanffy equation was used with two separate growth coefficients: LA = L∞ [1 − *e*^−*k*(*A* − *t*_0_^^)^], where LA is length at age, L∞ is theoretical maximum length of fish, *k* is growth coefficient, and *t*_0_ is theoretical age when length is 0 mm. After 30 min of thawing the fish, a cotton swab (Puritan; catalog number 806-wc) was swiped back and forth five times along the left gill and then put directly into a 2-ml MoBio PowerSoil (MoBio; catalog number 12888) bead beating tube. The skin was also swabbed in a 3-cm by 3-cm area on the left side behind the gill and above the pectoral fin (Fig. 1d). After carefully dissecting the fish with a new razor blade, the last 3 cm of GI tract was cleared and the digesta sampled. The same distal portion of GI tract was cut and also sampled. Lastly, an approximately 50-mg sample of pyloric ceca was sampled from the fish and placed in a tube. The tubes were then stored at –80°C until DNA extraction. For seawater samples from the pier, 400 μl of seawater was processed directly through DNA extraction. We chose not to filter to enable a more accurate volumetric or biomass comparison to the fish. Along with the time series from the pier, we also collected seawater and marine sediment from other locales in San Diego. These additional environmental controls, surface seawater and sediment samples, were collected across two time points (8 December 2017 and 12 January 2018) at 30 coastal locations, approximately 200 m offshore, at a 10-m depth, spanning 10 km throughout San Diego, including soft bottom, reef, river mouth, and bay areas. Sediment was collected using a grab mechanism made by Bill Fenical which grabs about 50 g of the surface sediment down to a 1- to 2-cm depth. Furthermore, in addition to the additional sediment and seawater samples, we also collected a total of 16 fish species from the local San Diego reefs, including 2 species of Embiotocidae (black surfperch [Embiotoca jacksoni] and walleye surfperch [Hyperprosopon argenteum]), 3 species of Kyphosidae (zebraperch [Hermosilla azure], opaleye [Girella nigricans], and half-moon [Medialuna californiensis]), 2 species of Labridae (senorita [Oxyjulis californica] and rock wrasse [Halichoeres semicinctus]), 1 Clinidae (kelp fish), 1 Serranidae (barred sand bass [Paralabrax nebulifer]), 1 Atherinidae, 2 Haemulidae (California salema [Xenistius californiensis] and sargo [Anisotremus davisonii]), 1 Clupeidae (sardine [Sardinops sagax]), 1 Carangidae (jack mackerel [Trachurus symmetricus]), 1 Synodontidae (lizard fish [Synodus lucioceps]), and 1 Pomacentridae (blacksmith [Chromis punctpinnis]).

### Microbiome processing.

Samples were processed using the standard 16S rRNA gene Earth Microbiome Protocol (EMP), with only slight modifications (http://www.earthmicrobiome.org). Specifically, genomic DNA was extracted using a hybrid approach in which lysis is performed in 2-ml bead beating tubes and then cleanup performed using the KingFisher robot to reduce well-to-well contamination (66). The initial cell lysis steps were performed in single-tube reactions (instead of a 96-well plate format) followed by transfer to plates for the standard magnetic bead cleanup on the KingFisher robots using the MoBio PowerMag kit (MoBio; catalog number 27000-4-KF), which has improved limits of detection for low-biomass samples (23). The EMP extraction procedure has modifications including the use of RNase A during lysis and a 10-min incubation at 65°C prior to bead beating. All sample batches had positive and negative controls included with each extraction set so that sample exclusions based on read counts could be calculated (23). Extracted gDNA was then PCR amplified using the EMP 16S V4 515f/806rB bar-coded primers (67, 68). The miniaturized PCR method, which generates libraries at a 58% cost reduction of $1.42 per sample, was used for all samples that included the use of the Echo-550 instrument to do triplicate 5-μl PCRs (69). Amplicons were quantified using a Pico green assay, and then 2 μl of each sample was equally pooled into a single tube. This final pool was then cleaned up to remove deoxynucleoside triphosphates (dNTPs) and primer dimers using the QIAquick PCR purification kit (Qiagen; catalog number 28106). Final pools contained up to 768 samples which were then sequenced on an Illumina MiSeq using a 2 by 150-bp strategy (300-cycle v2 kit; Illumina, San Diego, CA). Bioinformatic processing of samples was done using Qiita (70) and QIIME 2.0 (71), with the first 150-bp read trimmed to 150 bp and processed through deblur (72), a *de novo* sOTU picking method. A phylogenetic tree of the 16S sOTU single-sequence tags was created using SEPP (SATé-enabled phylogenetic placement) (73). Rarefaction levels were empirically determined by calculating the read counts at which 90% of the reads from the DNA extraction positive controls mapped back to the positive controls (23).

### Summary microbiome statistics.

Alpha, beta, and gamma diversity of microbial communities was measured (74). Alpha diversity was calculated using measures of Shannon diversity (75) and Faith’s phylogenetic diversity (76), while beta diversity was calculated using weighted and unweighted UniFrac (77, 78) distance and visualized in Emperor (79). Alpha and beta diversity statistical significance was tested using Kruskal-Wallis test (80). Taxonomies were classified in Silva (81) using the Greengenes and RDP databases (82) using the following parameters: minimum identity with query sequence (0.95), number of neighbors per query sequence (10), Greengenes reference NR database, search kmer-candidates (1,000), lca-quorum (0.8), search-kmer-length (10), search-kmer-mm (0), search-no-fast, and reject sequences below 70%.

### Statistical analysis of environmental and biological drivers of fish mucosal microbiomes.

To evaluate the extent to which the environment and biology of the fish influences the microbial communities of the various body sites, both alpha diversity and beta diversity were analyzed. Only samples which had environmental values for all water conditions (temperature, salinity, pressure, and chlorophyll *a*) and biological conditions (weight, fork length, condition factor, and age) were included. Thus, some samples had to be excluded due to temporary failure of the chlorophyll *a* fluorometer instrument on the SIO Pier (4 August 2017). Alpha diversity measures for each body site were independently verified and tested to ensure that they met the assumptions for the general linear model (GLM). Specifically, to test for normally distributed residuals, sets were analyzed using the R package Library(car) (83) and run through the Shapiro-Wilk normality test (84). To evaluate and test for homoscedasticity, the nonconstant error variance test (ncvTest) commonly known as the Breusch-Pagan test was used (85). To meet GLM criteria, the Faith’s phylogenetic diversity samples were log transformed. Both Shannon diversity and Faith’s PD were then processed through the GLM in R while controlling for collinearity of variables. Individual *R*^2^ values and *P* values for each environmental and biological variable are reported along with total *R*^2^, F-statistic, and *P* values for all variables. Gill samples using Shannon diversity (residual normality < 0.05, Shapiro test; homoscedasticity < 0.05, Breusch-Pagan test) and GI samples using Faith’s PD (residual normality < 0.05, Shapiro test) were excluded from analysis due to not meeting required assumptions of the GLM. To evaluate the effects of environmental and biological variables on beta diversity, we assessed both unweighted and weighted UniFrac for each body site independently using Adonis, a nonparametric analysis of variation method (86), in QIIME2 (71) and Calypso (87).

### Validation of Photobacterium damselae sOTU phylogeny.

To validate the taxonomy assignments of five *Photobacterium* sOTUs in our data set, we performed multiple-sequence alignment (neighbor joining) of a 148-bp region of the 16S rRNA gene v4 region from eight other strains. Specifically, we used default settings (nucleotide scoring 200 PAM/k = 2, gap opening penalty = 1.53, offset value = 0, “nzero” where N’s have no effect on alignment score) in the MAFFT alignment tool (88, 89). The phylogenetic tree was visualized using Phylo.io (90). The comparison bacteria strains included the following: two pathogenic *Photobacterium* species isolates (P. damselae ATCC 33539^T^, GenBank accession no. X74700.1; P. damselae, GenBank accession no. D25308.1), four nonpathogenic *Photobacterium* spp. (*P. leiognathi* strain ATCC 25521, GenBank accession no. NR_115541.1; *P. angustum* ATCC 25915^T^, GenBank accession no. X74685.1; *P. phosphoreum* strain ATCC 11040, GenBank accession no. NR_115205.1; *P. rosenbergii* strain CC1, GenBank accession no. NR_042343.1), and two outgroup *Vibrio* species (Vibrio pelagius strain ATCC 25916, GenBank accession no. NR_119059.1; Aliivibrio fischeri strain ATCC 7744, GenBank accession no. NR_115204.1) which were identified from various studies (91–93).

### Population genetics of *S. japonicus*.

Mitochondrial genes have been used for animal, particularly aquatic animal, population genetics for decades (94–99), whereas full mitogenome sequencing has become common only in the past 5 years (100). We developed a high-throughput two-fragment, mitochondrial amplicon workflow for the Oxford Nanopore Technologies long-read sequencer, which to our knowledge is the first demonstration of using ONT for this application with fish. Mitogenome assembly from whole-genome sequencing has been done on lobsters using ONT technology (101). A total of 96 gDNA skin mucus samples, spanning 5 collection months (27 August 2017 to 26 January 2018), were amplified in 192 separate 10-μl PCRs using 1 μl of gDNA, 5 μl of New England BioLabs (NEB) Long Amp mastermix (NEB; catalog number M0287S), and 3.4 μl of molecular-grade water and one of two primer combinations. The first mitochondrial DNA fragment (96 PCRs) used 0.4 μl of 10 μM forward primer (SJ_F1_655: 5′-TTT CTG TTG GTG CTG ATA TTG | CAA ACC TCA CCC TCC CTT GTT-3′) and 0.4 μl of 10 μM reverse primer (SJ_R1_7653: 5′-ACT TGC CTG TCG CTC TAT CTT | CAC CAC TAT TCG GTG GTC TGC-0.3′). The second fragment (96 PCRs) used 0.4 μl of 10 μM forward primer (SJ_F2_7425: 5′-TTT CTG TTG GTG CTG ATA TTG | CTC CCT GCC GTC ATT CTT ATC) and 0.4 μl of 10 μM reverse primer (SJ_R2_15424: 5′-ACT TGC CTG TCG CTC TAT CTT | CGA CGA CTA CGT CTG CGA CAA). All primers have ONT adaptor regions on the first 27 bases as indicated by “|.” All PCRs followed the following protocol: 94°C for 3 min, 25 cycles of 94°C for 30 s, 60°C for 30 s, 65°C for 8 min 20 s, and a final extension of 68°C for 10 min, followed by storage at 4°C. Following the first PCR, a second 5-μl PCR was conducted for each of the 96 samples by combining 2.5 μl of NEB Long Amp mastermix and 0.1 μl of a unique barcode (Oxford Nanopore PCR barcode kit 01–96, batch DK601001 brown box) and finally pooling 1.2 μl of each PCR product (first plus second fragment). Barcodes were transferred to the PCR plate using the acoustic liquid handler Labcyte Echo 550. The same PCR was used. A final 2 μl of sample was pulled from all samples and processed through the QiaQuick PCR purification kit (Qiagen; catalog number 28106) and run on a 1% agarose gel to confirm size. The pool was then run on a used MinION using the one-dimensional (1D) PCR barcoding protocol (SQK-LSK109). Samples were demultiplexed, uploaded to Galaxy (102), and aligned against the *Scomber japonicus* reference mitochondrial genome (GenBank accession no. NC_013723) using LASTZ aligner (Galaxy version 1.3.2) (103) using defaults. Consensus sequences were visualized, calculated, and exported using the quick consensus mode in Integrated Genome Viewer (104). Samples with less than an average of 10× coverage and samples with more than 20 ambiguous base pairs (N’s) were excluded from the analysis (*n* = 2; BC52 and BC82). A phylogenetic tree of all 91 *S. japonicus* samples along with three reference *S. japonicus* mitochondrial genomes from NCBI (GenBank accession no. AB488405.1, NC_013723.1, and AB102724.1), and two outgroup species, Scomber colias (NC_013724.1) and Scomber australasicus (AB102725.1), was generated using MAFFT (89) (NJ conserved sites 12388, Jukes-Cantor substitution model, bootstrap 100) and visualized with Phylo.io using default parameters (90).

All microbiome data are publicly available through Qiita (sample ID 11721, prep ID 4638), EBI (ERP109537), and NCBI (BioProject PRJEB27458).
