# Supplementary figures and images for: Temporal, Environmental, and Biological Drivers of the Mucosal Microbiome in a Wild Marine Fish, Scomber japonicus
Source: mSphere. 2020 May 20;5(3):e00401-20. doi: 10.1128/mSphere.00401-20 (PMC7380571; doi:10.1128/mSphere.00401-20)

Composition of target  
(*V. fischeri* or ZMC )

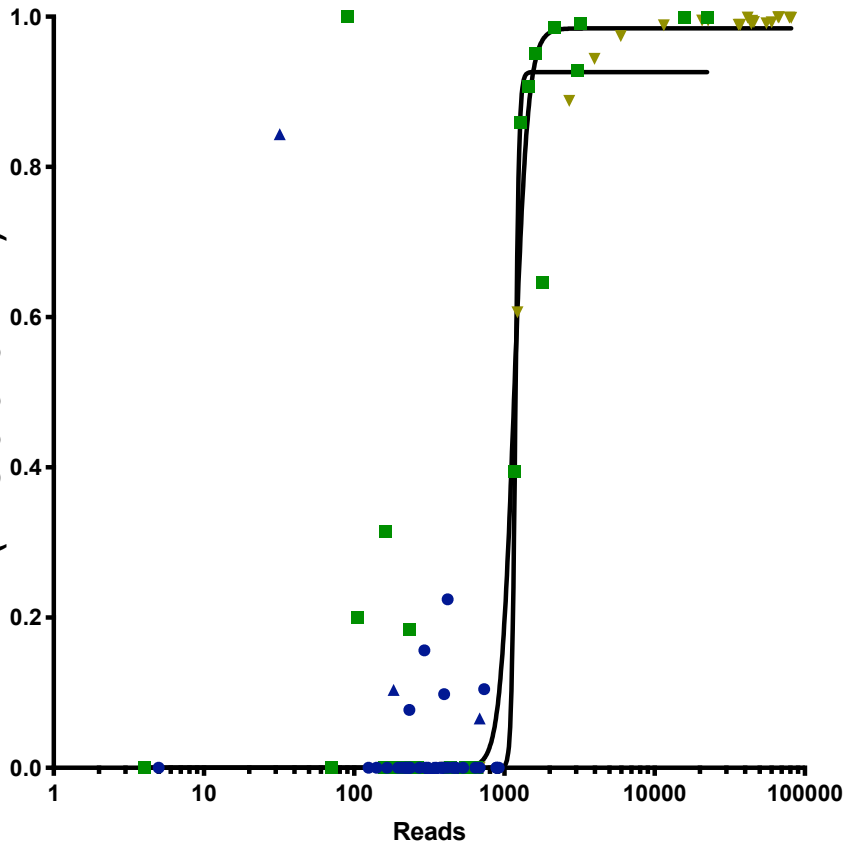

- DNA-
- DNA+
- ▲ PCR-
- ▼ PCR+ Vf or ZMC

Supplement: FIG S1 [file mSphere.00401-20-sf001.pdf]

s1

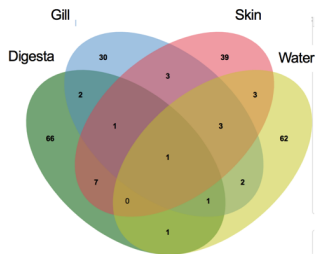

s3

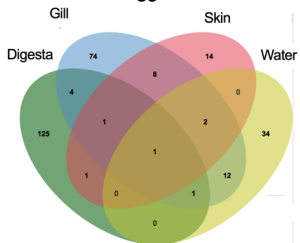

s5

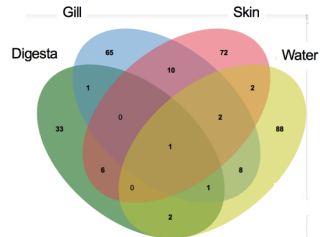

s9

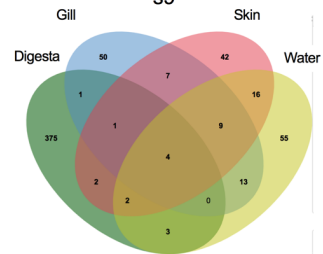

s12

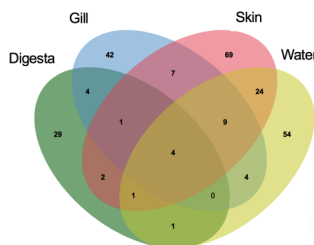

s15

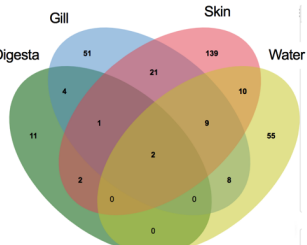

s17

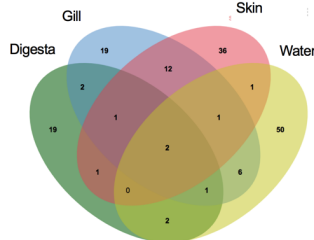

s20

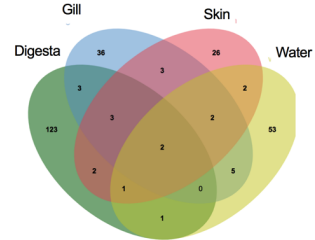

s34

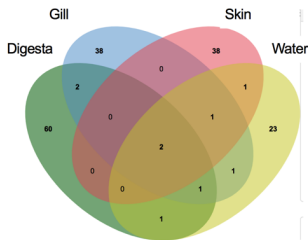

s38

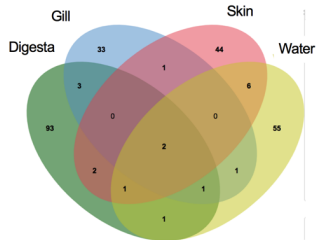

Supplement: FIG S2 [file mSphere.00401-20-sf002.pdf]

**a**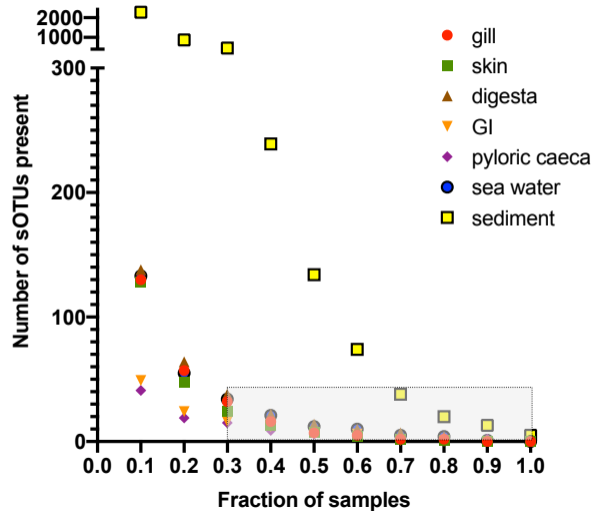**b**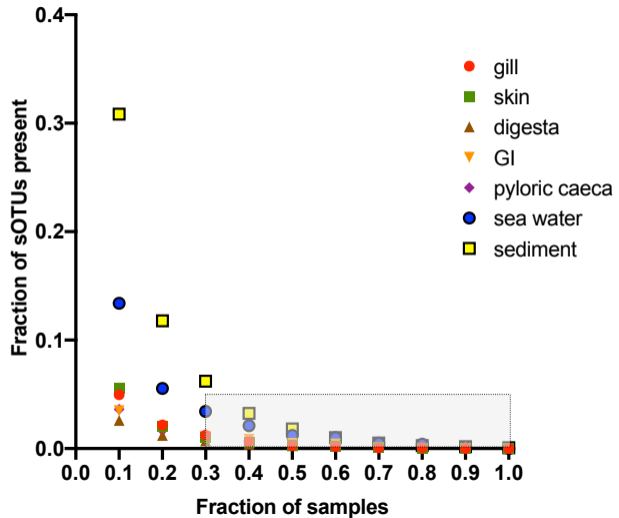

Supplement: FIG S3 [file mSphere.00401-20-sf003.pdf]

a

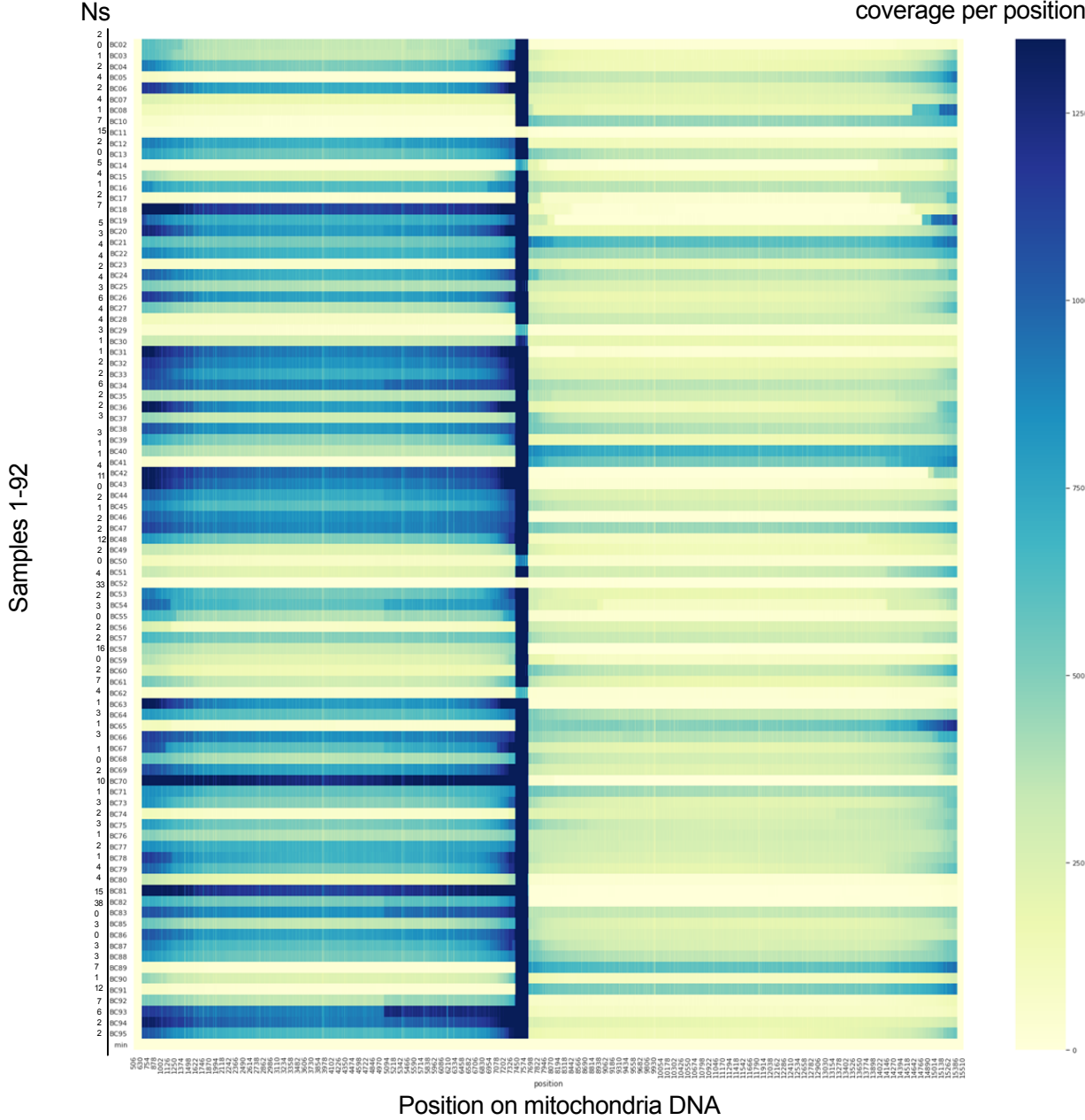

b

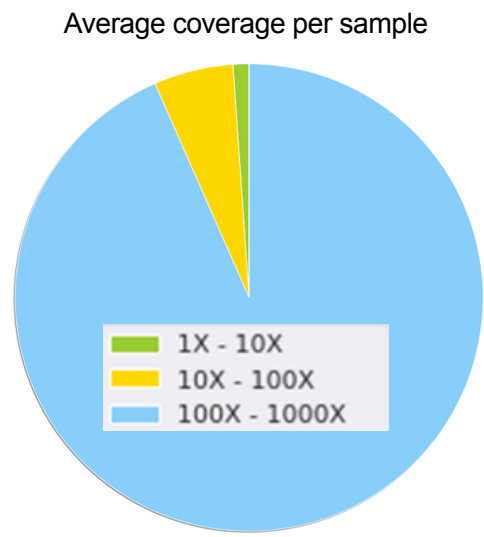

Supplement: FIG S4 [file mSphere.00401-20-sf004.pdf]

# No genetic population structure in of *S. japonicus* from 2017

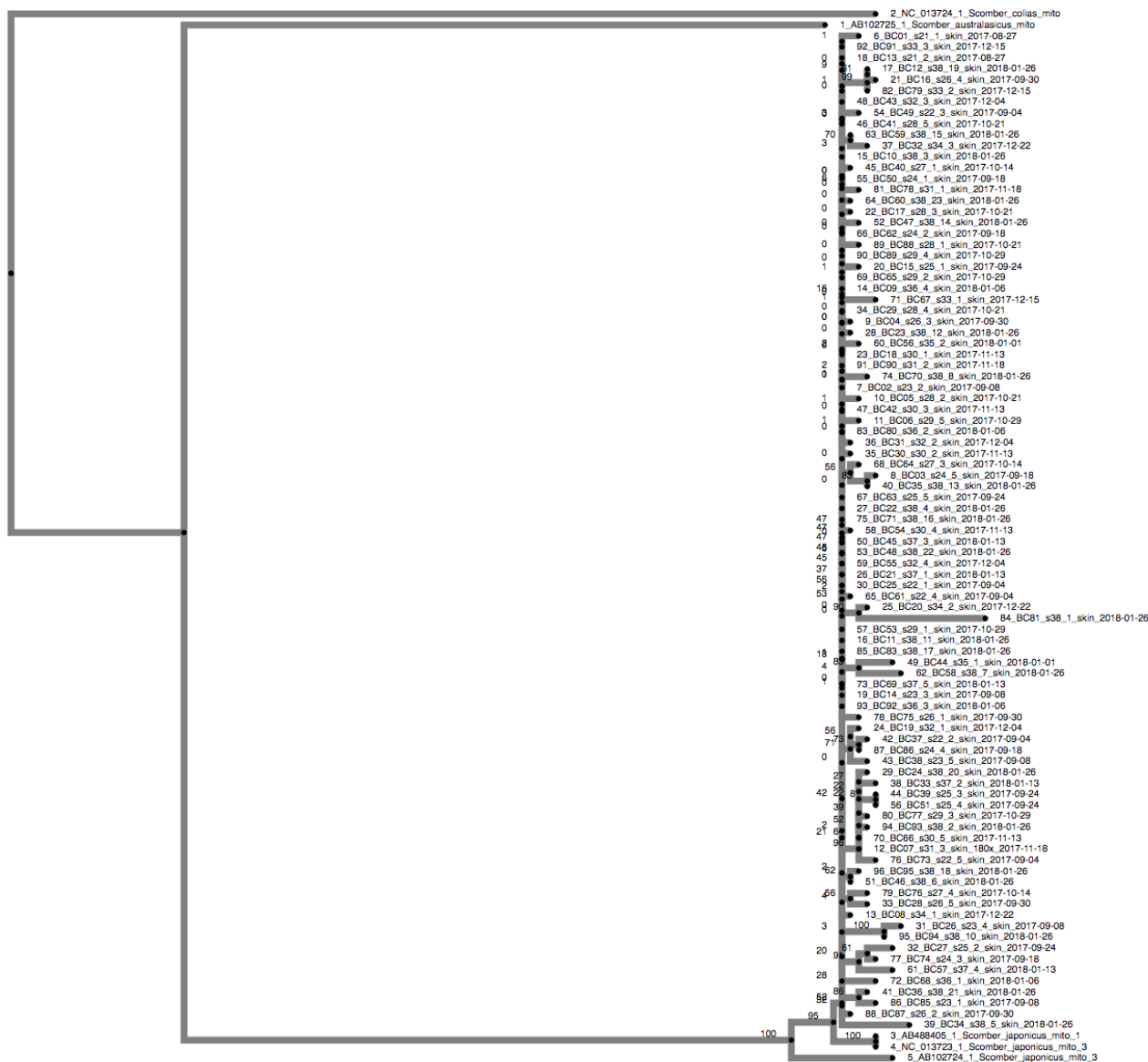

Supplement: FIG S5 [file mSphere.00401-20-sf005.pdf]
