# Supplementary material for: Temporal, Environmental, and Biological Drivers of the Mucosal Microbiome in a Wild Marine Fish, Scomber japonicus
Source: mSphere. 2020 May 20;5(3):e00401-20. doi: 10.1128/mSphere.00401-20 (PMC7380571; doi:10.1128/mSphere.00401-20)
Supplement: FIG S6 [file mSphere.00401-20-sf006.pdf]

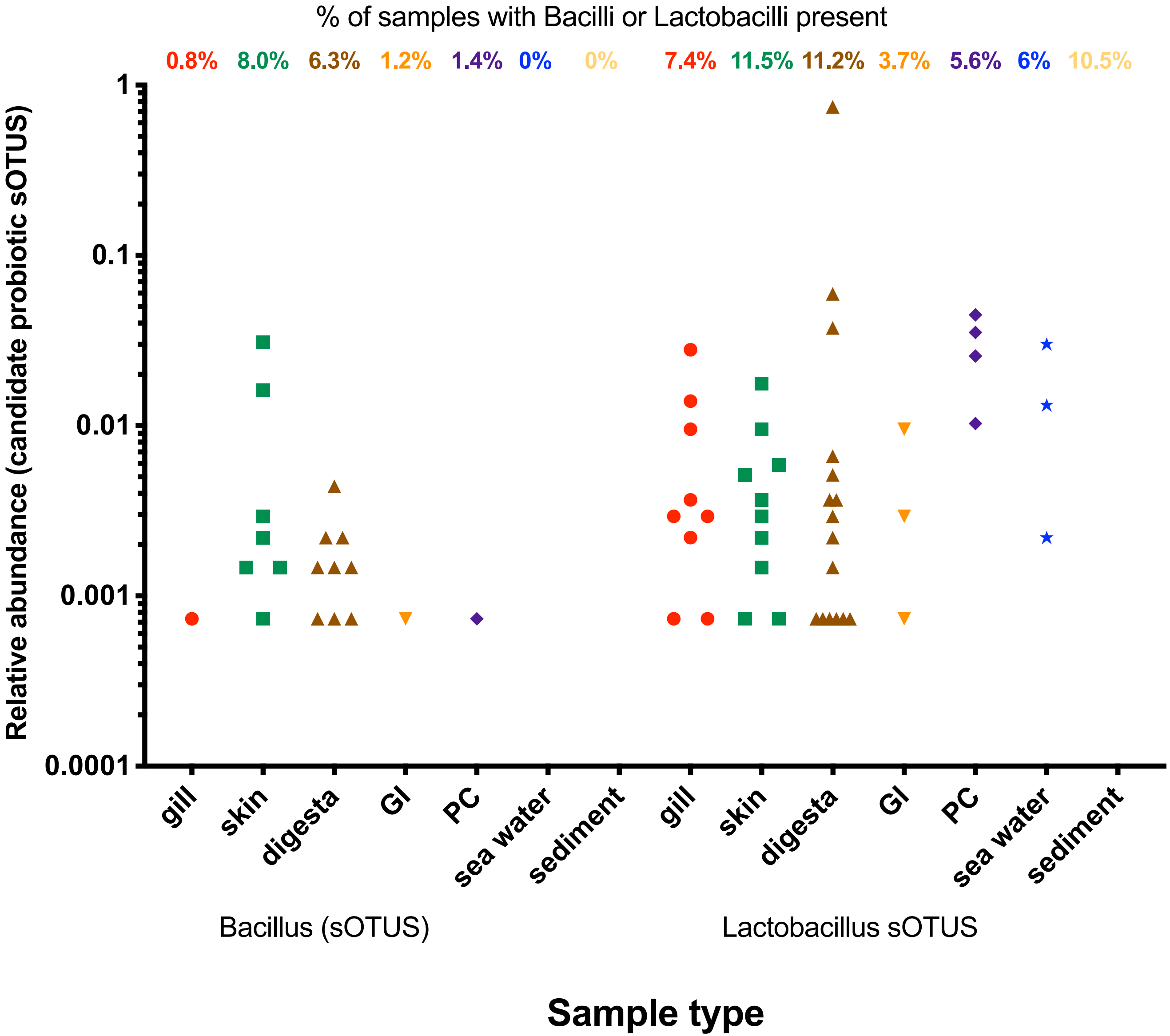
% of samples with Bacilli or Lactobacilli present
0.8%
8.0%
6.3%
1.2%
1.4%
0%
0%
7.4%
11.5%
11.2%
3.7%
5.6%
6%
10.5%
Relative abundance (candidate probiotic sOTUS)
1
0.1
0.01
0.001
0.0001
gill
skin
digesta
GI
PC
sea water
sediment
gill
skin
digesta
GI
PC
sea water
sediment
Bacillus (sOTUS)
Lactobacillus sOTUS
Sample type
